# Multidrug-resistant *Staphylococcus cohnii* and *Staphylococcus urealyticus* isolates from German dairy farms exhibit resistance to beta-lactam antibiotics and divergent penicillin-binding proteins

**DOI:** 10.1038/s41598-021-85461-6

**Published:** 2021-03-16

**Authors:** Tobias Lienen, Arne Schnitt, Jens Andre Hammerl, Stephen F. Marino, Sven Maurischat, Bernd-Alois Tenhagen

**Affiliations:** Department Biological Safety, German Federal Institute for Risk Assessment (BfR), 10589 Berlin, Germany

**Keywords:** Antimicrobial resistance, Bacterial genes

## Abstract

Non-*aureus* staphylococci are commonly found on dairy farms. Two rarely investigated species are *Staphylococcus* (*S*.) *cohnii* and *S*. *urealyticus*. Since multidrug-resistant *S. cohnii* and *S*. *urealyticus* are known, they may serve as an antimicrobial resistance (AMR) gene reservoir for harmful staphylococcal species. In our study, nine *S*. *cohnii* and six *S*. *urealyticus* isolates from German dairy farms were analyzed by whole-genome sequencing and AMR testing. The isolates harbored various AMR genes (*aadD1*, *str*, *mecA*, *dfrC/K*, *tetK/L*, *ermC*, *lnuA*, *fexA*, *fusF*, *fosB6*, *qacG/H*) and exhibited non-wildtype phenotypes (resistances) against chloramphenicol, clindamycin, erythromycin, fusidic acid, rifampicin, streptomycin, tetracycline, tiamulin and trimethoprim. Although 14/15 isolates lacked the *blaZ*, *mecA* and *mecC* genes, they showed reduced susceptibility to a number of beta-lactam antibiotics including cefoxitin (MIC 4–8 mg/L) and penicillin (MIC 0.25–0.5 mg/L). The specificity of cefoxitin susceptibility testing for *mecA* or *mecC* gene prediction in *S*. *cohnii* and *S*. *urealyticus* seems to be low. A comparison with penicillin-binding protein (PBP) amino acid sequences of *S. aureus* showed identities of only 70–80% with regard to PBP1, PBP2 and PBP3. In conclusion, *S*. *cohnii* and *S*. *urealyticus* from selected German dairy farms show multiple resistances to antimicrobial substances and may carry unknown antimicrobial resistance determinants.

## Introduction

Non-*aureus* staphylococci (NAS) are frequently found on dairy farms and occasionally cause intramammary infections^1–3^. Among others, *Staphylococcus* (*S*.) *cohnii* was found on farms; however, it has been regarded a commensal bacterium, and not involved in severe animal infections such as bovine mastitis^2,4^. *S*. *cohnii* has been divided into the subspecies *S*. *cohnii* subsp. *cohnii* and *S*. *cohnii* subsp. *urealyticus*. However, recent phylogenetic studies suggested that the subspecies *S*. *cohnii* subsp. *urealyticus* should be regarded as an individual species *S*. *urealyticus*^5^. With respect to human health, *S*. *cohnii* has been reported to be an opportunistic pathogen^6,7^. Multidrug-resistant *S. cohnii* isolates were recently detected on frequently touched surfaces in a London hospital^8^. Accordingly, *S*. *cohnii* as well as *S*. *urealyticus* isolates of animal origin were shown to carry multiple antimicrobial resistance (AMR) genes and express phenotypic resistance to various classes of antimicrobials^9–11^. Even resistance to last resort antibiotics such as linezolid was demonstrated^12^. Thus far, only five complete genomes of *S*. *cohnii* or *S*. *urealyticus* are available in public databases such as NCBI Genome, complicating comparison of alterations in gene function with regard to AMR. Novel resistance determinants such as the fusidic acid resistance gene *fusF* have been discovered in *S. cohnii* isolates^13^. The most prominent resistance gene in staphylococci is *mecA*, which encodes a variant penicillin-binding protein (PBP) 2a. A PBP is a transpeptidase, which is crucial for bacterial cell wall synthesis. Beta-lactam antibiotics bind to the PBP and thus inhibit the transpeptidase reaction. Differences in the structure of PBP2a compared to other PBPs result in a lower binding affinity for beta-lactam antibiotics, causing resistance^14,15^. The *mecA* gene is located on the staphylococcal cassette chromosome (SCC) *mec* element, which has been categorized into 13 types^16,17^. It was shown that NAS may harbor high diversity and unknown SCC*mec* elements^8,18^ and new SCC*mec* elements have indeed been described for *S*. *cohnii*^6,19^. Determination of beta-lactam antibiotic resistance in *S*. *aureus*, a well investigated staphylococcal species, and the identification of methicillin-resistant *S*. *aureus* (MRSA) is routinely performed by cefoxitin resistance testing and detection of the *mecA* or *mecC* gene. However, several studies have reported that the determination of beta-lactam antibiotic resistance in NAS using cefoxitin susceptibility testing might be misleading and the use of oxacillin should be favored^20–22^.

The aim of our study was to evaluate the antimicrobial resistance potential of *S*. *cohnii* and *S*. *urealyticus* isolates from German dairy farms. The isolates were analyzed by whole-genome sequencing (WGS) and AMR testing. AMR gene prediction was linked to the respective phenotypic resistance pattern. A special focus was set on beta-lactam antibiotic resistance and related AMR genes, since beta-lactams are commonly used to treat infections such as mastitis in livestock. Since NAS might serve as an AMR gene reservoir for *S. aureus*, which is a major animal and human pathogenic species, monitoring of multidrug-resistant NAS is of high importance.

## Results

### Phylogenetic analysis

**Figure 1:**
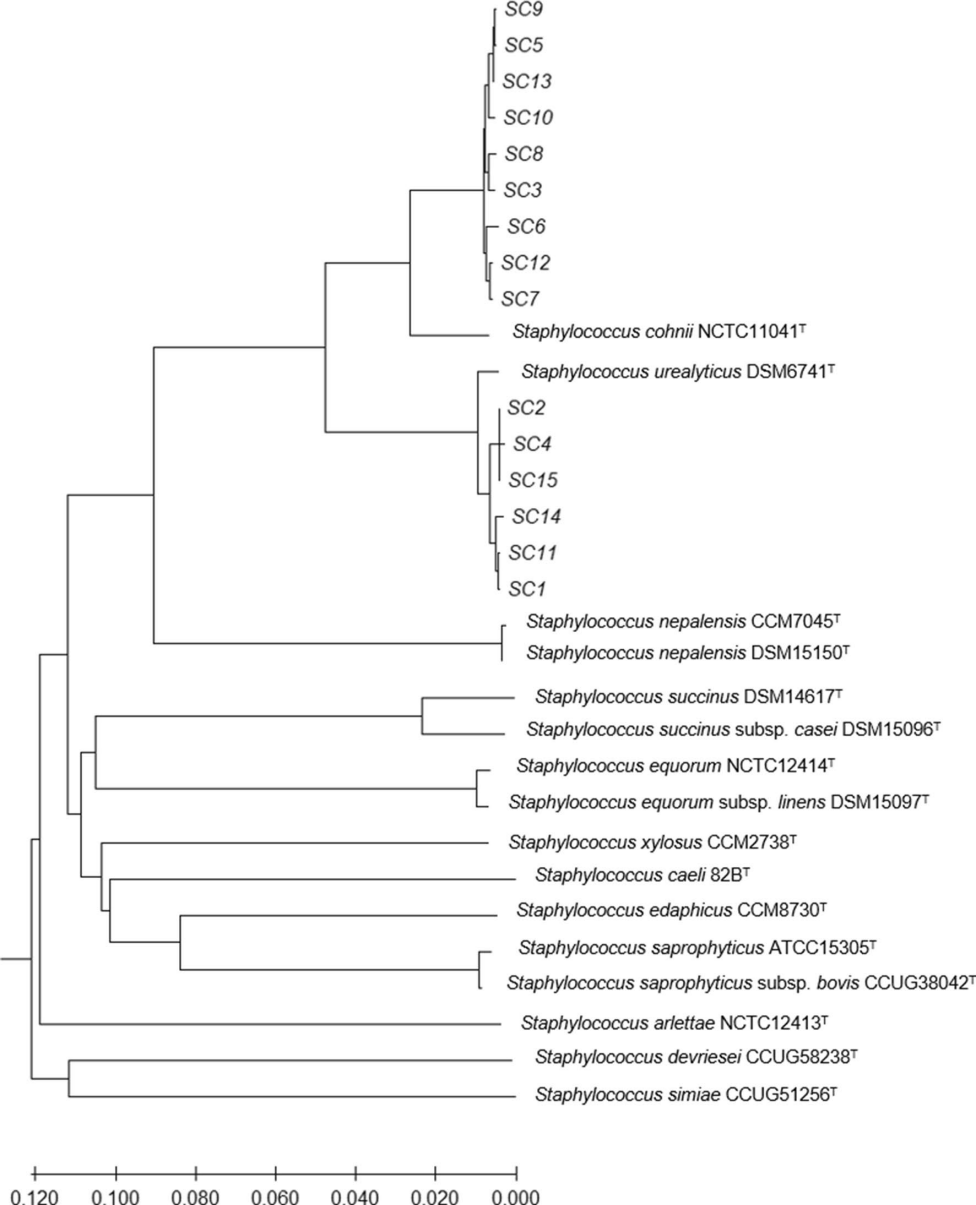
Phylogenetic analysis by TYGS of 15 presumptive *S. cohnii* isolates from German dairy farms reveals two main clusters of relationship. Clustering of isolates SC1, SC2, SC4, SC11, SC14 and SC15 together with *S*. *urealyticus* DSM6718 indicates assignment of these isolates to the species.

Fifteen staphylococcal isolates from German dairy farms were presumptively identified as *S*. *cohnii* by MALDI-TOF-MS analysis. According to the phylogenetic analysis using the type genome server (TYGS), the isolates SC3, SC5, SC6, SC7, SC8, SC9, SC10, SC12 and SC13 were assigned to a *S*. *cohnii* type strain, while SC1, SC2, SC4, SC11, SC14 and SC15 clustered close to the type strain *S*. *cohnii* subsp. *urealyticus* DSM 6718 (Fig. 1). Recent phylogenetic analyses suggest the promotion of *S*. *cohnii* subsp. *urealyticus* to the novel species *S*. *urealyticus*^5^. Moreover, the genotypical differences between *S*. *cohnii* isolates within one farm were determined by comparing two different isolates from the same farm. Isolates SC7/8 and SC9/10 were therefore assigned to the same cluster, respectively.

### AMR genes

The isolates harbored various AMR genes with a variable distribution among the isolates. Resistance to the antibiotic classes aminoglycosides (*aadD1*, *str*), beta-lactams (*mecA*), trimethoprim (*dfrC/K*), tetracyclines (*tetK/L*), macrolides (*ermC*), pleuromutilin–lincosamide–streptogramin (*lnuA*), phenicols (*fexA*), fusidic acid (*fusF*), fosfomycin (*fosB6*) and to quaternary ammonium compounds (*qacG/H*) was predicted. The number of detected AMR genes per isolate varied between eight (1/15), seven (4/15), five (1/15), four (3/15) and three (6/15).

The *fusF* and *dfrC* genes were detected in all *S*. *cohnii* and *S*. *urealyticus* isolates (Table 1). The *lnuA* (2/9 *S*. *cohnii* isolates), *aadD1* (2/6 *S*. *urealyticus* isolates), *ermC* (1/9 *S*. *cohnii* isolates; 1/6 *S*. *urealyticus* isolates), *tetL* (2/6 *S*. *urealyticus* isolates), *mphC* (4/9 *S*. *cohnii* isolates), *str* (3/9 *S*. *cohnii* isolates; 1/6 *S*. *urealyticus* isolates), *tetK* (4/9 *S*. *cohnii* isolates; 1/6 *S*. *urealyticus* isolates), *qacH* (6/6 *S*. *urealyticus* isolates) and *msrA* (9/9 *S*. *cohnii* isolates) genes were only detected in some isolates or in only one of the two species. In contrast, the *mecA* (SC7, *S*. *cohnii*), *fexA* (SC4, *S*. *urealyticus*), *dfrK* (SC4, *S*. *urealyticus*), *fosB6* (SC9, *S*. *cohnii*) and *qacG* (SC15, *S*. *urealyticus*) genes were each present in only one isolate. The *mecA* gene of isolate SC7 was located on a SCC*mec* type V.

**Table 1:** Predicted antimicrobial resistance genes compared to results of minimum inhibitory concentration of various antimicrobial substances for the *S. cohnii* and *S*. *urealyticus* isolates SC1-15 from German dairy farms.

Strain	Species	Farm	AMR^1^ genes	MIC^2,3^ (mg/L)																
				CHL	CIP	CLI	ERY	FUS	GEN	KAN	LZD	MUP	RIF	SMX	STR	SYN	TET	TIA	TMP	VAN
SC1	*S. urealyticus*	1	*dfrC; fusF; qacH*	8	1	1	≤ 0.25	> 4	≤ 1	≤ 4	≤ 1	≤ 0.5	0.06	≤ 64	≤ 4	≤ 0.5	1	> 4	≤ 2	2
SC2	*S. urealyticus*	2	*dfrC; fusF; qacH*	≤ 4	0.5	0.25	≤ 0.25	> 4	≤ 1	≤ 4	≤ 1	≤ 0.5	0.03	≤ 64	≤ 4	≤ 0.5	≤ 0.5	4	≤ 2	≤ 1
SC3	*S. cohnii*	3	*dfrC; fusF; msr(A)*	8	0.5	0.5	> 8	> 4	≤ 1	≤ 4	≤ 1	≤ 0.5	0.5	128	≤ 4	≤ 0.5	1	> 4	4	2
SC4	*S. urealyticus*	4	*aadD1; dfrC; dfrK; fexA; fusF; qacH; str; tet(L)*	64	≤ 0.25	0.5	≤ 0.25	> 4	≤ 1	8	≤ 1	≤ 0.5	0.03	≤ 64	> 32	≤ 0.5	> 16	4	> 32	≤ 1
SC5	*S. cohnii*	5	*dfrC; fusF; msr(A)*	8	0.5	≤ 0.12	2	> 4	≤ 1	≤ 4	≤ 1	≤ 0.5	0.12	≤ 64	≤ 4	≤ 0.5	≤ 0.5	1	≤ 2	≤ 1
SC6	*S. cohnii*	6	*dfrC; fusF; mph(C); msr(A)*	≤ 4	0.5	0.25	4	> 4	≤ 1	≤ 4	≤ 1	≤ 0.5	0.25	≤ 64	≤ 4	1	≤ 0.5	2	≤ 2	2
SC7	*S. cohnii*	7	*dfrC; fusF; mecA; mph(C); msr(A); str; tet(K)*	16	≤ 0.25	0.25	2	> 4	≤ 1	≤ 4	≤ 1	≤ 0.5	0.5	≤ 64	> 32	1	> 16	> 4	≤ 2	2
SC8	*S. cohnii*	7	*dfrC; fusF; msr(A)*	16	1	0.5	> 8	> 4	≤ 1	≤ 4	≤ 1	≤ 0.5	0.5	≤ 64	≤ 4	≤ 0.5	1	> 4	4	≤ 1
SC9	*S. cohnii*	8	*dfrC; fosB6; fusF; lnu(A); msr(A)*	≤ 4	≤ 0.25	0.5	2	> 4	≤ 1	≤ 4	≤ 1	≤ 0.5	0.25	≤ 64	≤ 4	≤ 0.5	≤ 0.5	> 4	≤ 2	≤ 1
SC10	*S. cohnii*	8	*dfrC; fusF; lnu(A); mph(C); msr(A); str; tet(K)*	8	0.5	1	2	> 4	≤ 1	≤ 4	≤ 1	≤ 0.5	0.03	≤ 64	> 32	≤ 0.5	> 16	> 4	8	≤ 1
SC11	*S. urealyticus*	9	*aadD1; dfrC; erm(C); fusF; qacH; tet(K); tet(L)*	8	0.5	> 4	> 8	> 4	≤ 1	8	≤ 1	≤ 0.5	0.06	≤ 64	≤ 4	1	> 16	> 4	≤ 2	≤ 1
SC12	*S. cohnii*	9	*dfrC; erm(C); fusF; mph(C); msr(A); str; tet(K)*	≤ 4	≤ 0.25	> 4	> 8	1	≤ 1	≤ 4	≤ 1	≤ 0.5	0.12	≤ 64	≤ 4	≤ 0.5	16	≤ 0.5	≤ 2	≤ 1
SC13	*S. cohnii*	10	*dfrC; fusF; msr(A); tet(K)*	8	0.5	≤ 0.12	4	> 4	≤ 1	≤ 4	2	≤ 0.5	0.25	≤ 64	≤ 4	≤ 0.5	> 16	> 4	4	≤ 1
SC14	*S. urealyticus*	11	*dfrC; fusF; qacH*	≤ 4	0.5	0.25	≤ 0.25	> 4	≤ 1	≤ 4	≤ 1	≤ 0.5	0.03	≤ 64	≤ 4	≤ 0.5	≤ 0.5	4	≤ 2	≤ 1
SC15	*S. urealyticus*	12	*dfrC; fusF; qacG; qacH*	8	0.5	0.25	0.5	> 4	≤ 1	≤ 4	≤ 1	≤ 0.5	0.03	≤ 64	≤ 4	≤ 0.5	1	4	≤ 2	≤ 1
EUCAST ECOFF^5^			*Staphylococcus aureus*	≤ 16	≤ 1	≤ 0.25	≤ 1	≤ 0.5	≤ 2	≤ 8	≤ 4	≤ 1	≤ 0.016	≤ 128	≤ 16	≤ 1	≤ 1	≤ 2	≤ 2	≤ 2
			Coagulase negative staphylococci	≤ 16	≤ 1	≤ 0.25	≤ 1	≤ 0.5	ND^4^	ND	≤ 2	ND	≤ 0.064	ND	ND	ND	≤ 1	ND	ND	≤ 4

^1^*AMR *antimicrobial resistance, ^2^*MIC *minimum inhibitory concentration, ^3^*CHL *chloramphenicol, *CIP *ciprofloxacin, *CLI *clindamycin, *ERY *erythromycin, *FUS *fusidic acid, *GEN *gentamycin, *KAN *kanamycin, *LZD *linezolid, *MUP *mupirocin, *RIF *rifampicin, *SMX *sulfamethoxazole, *STR *streptomycin, *SYN *quinupristin-dalfopristin, *TET *tetracycline, *TIA *tiamulin, *TMP *trimethoprim, *VAN *vancomycin, ^4^*ND *not defined. ^5^Epidemiological cut-off values as provided by EUCAST (http://www.eucast.org; accessed August 31, 2020).

### Phenotypic AMR testing

The minimum inhibitory concentration (MIC) values for various antimicrobial substances are shown in Tables 1 and 2. Multidrug resistance to at least three antimicrobial classes was found in all isolates. According to the EUCAST epidemiological cut-off values (ECOFFs) for *S. aureus* and coagulase negative staphylococci, a non-wildtype phenotype was determined in several isolates for chloramphenicol (1/6 *S*. *urealyticus* isolates), clindamycin (5/9 *S*. *cohnii* isolates; 3/6 S. *urealyticus* isolates), erythromycin (9/9 *S*. *cohnii* isolates; 1/6 *S*. *urealyticus* isolates), fusidic acid (9/9 *S*. *cohnii* isolates; 6/6 *S*. *urealyticus* isolates), rifampicin (8/9 *S*. *cohnii* isolates), streptomycin (2/9 *S*. *cohnii* isolates; 1/6 *S*. *urealyticus* isolates), tetracycline (4/9 *S*. *cohnii* isolates; 2/6 *S*. *urealyticus* isolates), tiamulin (6/9 *S*. *cohnii* isolates; 6/6 *S*. *urealyticus* isolates) and trimethoprim (4/9 *S*. *cohnii* isolates; 1/6 *S*. *urealyticus* isolates) (Table 1).

**Table 2:** Predicted beta-lactam antimicrobial resistance genes and minimum inhibitory concentration of *S. cohnii* and *S*. *urealyticus* isolates SC1-15 from twelve German dairy farms.

Strain	Species	Farm	Beta-lactam AMR^1^ genes	MIC^2,3^ (mg/L)												
				FOX	OXA	PEN	AMP	FEP	ETP	IMP	MERO	FOT	FOTCLA	TAZ	TAZCLA	TEMOCI
SC1	*S. urealyticus*	1	*–*	8	0.5	0.5	≤ 1	4	2	0.25	0.5	8	8	32	32	> 128
SC2	*S. urealyticus*	2	*–*	8	0.5	0.25	≤ 1	4	2	≤ 0.12	0.5	8	8	32	32	> 128
SC3	*S. cohnii*	3	*–*	8	1	0.5	≤ 1	4	2	≤ 0.12	0.5	16	8	64	32	> 128
SC4	*S. urealyticus*	4	*–*	8	1	0.5	≤ 1	8	2	≤ 0.12	0.5	16	8	64	32	> 128
SC5	*S. cohnii*	5	*–*	8	1	0.5	≤ 1	4	2	≤ 0.12	0.5	8	8	64	32	> 128
SC6	*S. cohnii*	6	*–*	4	1	0.5	≤ 1	4	2	≤ 0.12	0.5	8	8	64	64	> 128
SC7	*S. cohnii*	7	*mecA*	> 16	> 8	> 2	4	> 32	> 2	0.25	2	> 64	64	128	> 128	> 128
SC8	*S. cohnii*	7	*–*	8	0.5	0.5	≤ 1	4	2	≤ 0.12	0.5	8	8	32	32	> 128
SC9	*S. cohnii*	8	*–*	4	0.5	0.25	≤ 1	2	2	≤ 0.12	0.25	4	4	32	16	> 128
SC10	*S. cohnii*	8	*–*	8	0.5	0.5	≤ 1	4	2	≤ 0.12	0.5	8	8	32	32	> 128
SC11	*S. urealyticus*	9	*–*	4	0.5	0.25	≤ 1	2	2	≤ 0.12	0.5	8	4	64	32	> 128
SC12	*S. cohnii*	9	*–*	8	0.5	0.25	≤ 1	2	2	≤ 0.12	0.5	4	4	32	16	> 128
SC13	*S. cohnii*	10	*–*	4	0.5	0.25	≤ 1	2	2	≤ 0.12	0.5	8	8	32	32	> 128
SC14	*S. urealyticus*	11	*–*	4	0.5	0.25	≤ 1	2	2	≤ 0.12	0.5	4	4	16	16	> 128
SC15	*S. urealyticus*	12	*–*	8	0.5	0.5	≤ 1	2	2	≤ 0.12	0.5	8	4	32	16	> 128
EUCAST ECOFF^5^			*Staphylococcus aureus*	≤ 4	≤ 2	≤ 0.125	ND	≤ 8	ND	≤ 0.125	≤ 0.5	≤ 4	ND	ND	ND	ND
			Coagulase negative staphylococci	ND^4^	≤ 1	ND	ND	ND	ND	≤ 0.125	≤ 0.5	≤ 2	ND	ND	ND	ND

^a^*AMR *antimicrobial resistance, ^b^*MIC *minimum inhibitory concentration, ^c^*FOX *cefoxitin, *OXA *oxacillin, *PEN *penicillin, *AMP *ampicillin, *FEP *cefepim, *ETP *ertapenem, *IMP* imipenem, *MERO *meropenem, *FOT *cefotaxime, *FOTCLA *cefotaxime/clavulanic acid, *TAZ *ceftazidime, *TAZCLA *ceftazidime/clavulanic acid, *TEMOCI *temocillin; ^4^*ND *not defined. ^5^Epidemiological cut-off values as provided by EUCAST (http://www.eucast.org; accessed December 7, 2020).

With regard to resistance to beta-lactam antibiotics, reduced susceptibilities were detected for penicillin (9/9 *S*. *cohnii* isolates; 6/6 *S*. *urealyticus* isolates), temocillin (9/9 *S*. *cohnii* isolates; 6/6 *S*. *urealyticus* isolates), cefoxitin (6/9 *S*. *cohnii* isolates; 4/6 *S*. *urealyticus* isolates), ceftazidime (9/9 *S*. *cohnii* isolates; 6/6 *S*. *urealyticus* isolates) and cefotaxime (7/9 *S*. *cohnii* isolates; 5/6 *S*. *urealyticus* isolates) (Table 2). The combination of cefotaxime or ceftazidime with the beta-lactamase inhibitor clavulanic acid only slightly lowered the MICs in four and seven isolates, respectively. With regard to cefotaxime, the MIC was lowered from 16 to 8 mg/L in one *S*. *cohnii* isolate and one *S*. *urealyticus* isolate, and from 8 to 4 mg/L in two more *S*. *urealyticus* isolates. Regarding ceftazidime, after addition of clavulanic acid a MIC reduction from 64 to 32 mg/L was detected in two *S*. *cohnii* and two *S*. *urealyticus* isolates, as well as from 32 to 16 mg/L in two *S*. *cohnii* isolates and one *S*. *urealyticus* isolate (Table 2). In contrast, all *mecA*/*mecC* negative isolates were susceptible to oxacillin, ampicillin, cefepime, meropenem, ertapenem and imipenem. The *mecA* gene harboring *S*. *cohnii* isolate SC7 exhibited a non-wildtype phenotype to all beta-lactam antibiotics.

### Beta-lactam AMR genes

With regard to beta-lactam antibiotic resistance, several genes of interest were analyzed. With the exception of *S*. *cohnii* isolate SC7, not only the *mecA* and *mecC* genes, but also *mecB* and *mecD* were absent from all genomes. In addition, there were no *femB*, *femC*, *femD*, *mgrA* or *mprF* genes which might be associated with beta-lactam resistance^23^. Only the *femA* and *gdpP* genes were detected.

**Figure 2:**
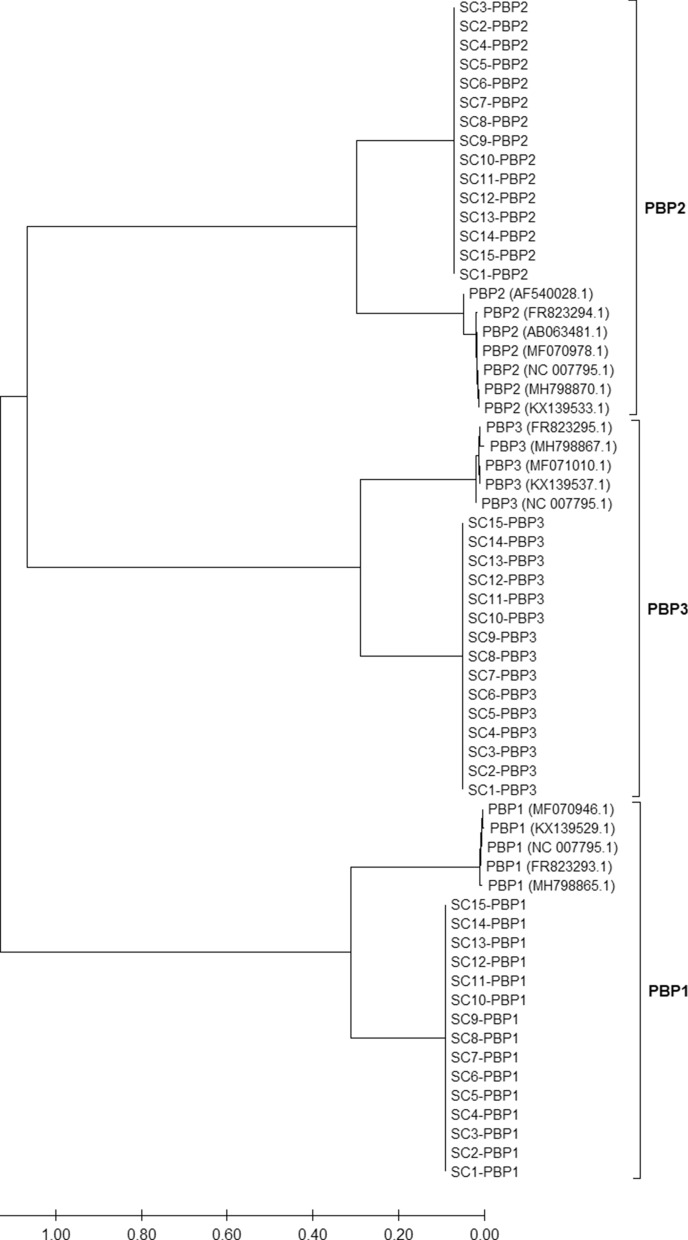
Neighbor-joining phylogenetic tree with reference *S. aureus* penicillin-binding protein (PBP) 1, PBP2 and PBP3 amino acid sequences in comparison to PBPs of the investigated *S. cohnii* and *S*. *urealyticus* isolates from German dairy farms. PBP1-3 genes were detected in each *S. cohnii* and *S*. *urealyticus* isolate. Amino acid sequence identity of PBPs from *S. aureus* and *S. cohnii*/*S*. *urealyticus* was only 70–80%.

Resistance to beta-lactam antibiotics might be mediated by the BlaZ beta-lactamase, which is encoded by the *blaZ* gene. However, the *blaZ* gene was not detected in any *S. cohnii* isolate in our study either using the NCBI amrfinder database or NCBI BLAST against known *blaZ* gene sequences. According to reference *S. cohnii* PBP gene sequences, all isolates from our study carried one PBP1, PBP2 and PBP3 gene each. Between isolates, the amino acid sequences of each respective PBP showed high identities, whereby the amino acid sequence identities between PBP1, 2 and 3 varied as also shown for the PBPs of *S. aureus* (Fig. 2). Interestingly, PBPs 1, 2 and 3 shared only approximately 70–80% amino acid sequence identity to the known PBP1, PBP2 and PBP3 of *S. aureus* (Fig. 2).

## Discussion

In our study, the phylogenetic relationship and antimicrobial resistance potential of nine *S. cohnii* and six *S*. *urealyticus* isolates from German dairy farms were analyzed. The staphylococcal species *S. cohnii* has been reported as a prevalent commensal bacterium on dairy farms that can be found on teat apices and in other milking parlor-related extramammary niches^4,24^. Previous studies suggested that *S. cohnii* is largely uninvolved in severe infections such as mastitis in dairy cows^2,4^. However, NAS may harbor a wide range of AMR genes and serve as a resistance gene reservoir for pathogenic species^9,10^.

The phylogenetic analysis revealed a clear genomic distinction from other NAS species. It is likely that isolates SC1, SC2, SC4, SC11, SC14 and SC15 belong to the subspecies *S*. *cohnii* subsp. *urealyticus*, which was recently declared to be a separate species *S*. *urealyticus*^5^, since these isolates clustered together with the respective type strain. Multidrug-resistant *S*. *cohnii* subsp. *urealyticus* (now *S*. *urealyticus*^5^) strains were also reported from a goat´s nasal cavity sample from Tanzania^11^ and from an ear swab of a healthy dog^25^.

*S*. *cohnii* isolates SC7 and SC8 as well as SC9 and SC10, each pair originating from one farm, grouped closely together, indicating the high clonality of these isolates. Thus, the spread of one *S*. *cohnii* clone within one farm seems likely. The transmission of staphylococci within dairy farms may be due to interaction of animals, farm workers and/or insufficient hygiene procedures during the milking process^26^.

The phenotypic resistance to fusidic acid in all *S*. *cohnii* and *S*. *urealyticus* isolates was in accordance with the detection of the *fusF* gene indicating its contribution to the resistant phenotype. Our study implies a wide distribution of this gene in *S. cohnii* and *S*. *urealyticus* isolates across the investigated German dairy farms. The *fusF* gene was originally detected in *S. cohnii* subsp. *urealyticus* (now *S*. *urealyticus*^5^) isolates and was suggested to be an intrinsic factor in this species^13^. Chloramphenicol resistance with a MIC value of 64 mg/L was determined for only one *S*. *urealyticus* isolate, SC4. This was the only isolate that harbored the *fexA* gene. This gene encodes a chloramphenicol/florfenicol efflux MFS transporter and was likely involved in the manifested resistance. In accordance with the low frequency of detection of the *fexA* gene in our study, a low *fexA* gene prevalence was also shown in NAS from Canadian dairy herds^10^ and low resistance rates to chloramphenicol were observed in MRSA from German dairy farms^27,28^.

*S*. *cohnii* isolate SC11 and *S*. *urealyticus* isolate SC12 showed increased MIC values for the lincosamide clindamycin (MIC > 4 mg/L) and the macrolide erythromycin (MIC > 8 mg/L). Both isolates carried the *ermC* gene, which most likely led to the resistance phenotype. The *ermC* gene is the most widespread *erm* gene in staphylococci and it is often located on plasmids^29^. A cross-resistance to macrolides and lincosamides in staphylococci mediated by constitutive expression of the *ermC* gene has been described in several studies^30,31^, and the mode of action of these antibiotics is very similar. Erythromycin resistance according to EUCAST ECOFFs was also detected in *S*. *cohnii* isolates SC3, SC5, SC6, SC7, SC8, SC9, SC10 and SC13. These isolates harbored the *msrA* gene, which encodes an ATP-binding protein that mediates erythromycin resistance^32^ and this AMR gene may have played a role in erythromycin resistance in our isolates. The *msrA* gene was also associated with erythromycin resistance in NAS isolates from dairy cattle^10^.

Resistance to the aminoglycoside streptomycin was found in *S*. *urealyticus* isolate SC4 and *S*. *cohnii* isolates SC7 and SC10. Accordingly, these isolates harbored the *str* gene, which mediates resistance to streptomycin. In contrast to studies which demonstrated resistance to several aminoglycosides such as gentamicin or kanamycin in MRSA isolates from dairy cows^28,33^ and NAS from veal calves^34^, the isolates in our study showed only resistance to streptomycin. However, reduced susceptibility to kanamycin (MIC 8 mg/L) was detected in *S*. *urealyticus* isolates SC4 and SC11. Both isolates carried the *aadD1* gene, which may mediate resistance to aminoglycosides such as tobramycin with a MIC ≥ 128 mg/L^35^, but apparently only leads to a reduced susceptibility to kanamycin.

Resistance to tetracycline was detected in four *S. cohnii* and two *S. urealyticus* isolates and in all cases was most likely mediated by the *tetL* and/or *tetK* genes. The *tetL* or *tetK* genes were also prevalent in NAS isolates from Canadian dairy herds^10^ and from Belgian veal calves^34^. Tetracyclines have been extensively used on animal farms, thus promoting the survival of tetracycline resistant isolates^36^. However, in comparison to previous studies with MRSA isolates from dairy farms, in which the tetracycline resistance prevalence reached 95–100%^28,33,37^, the 44% (4/9) or 33% (2/6) tetracycline resistance frequency of the *S. cohnii* and *S. urealyticus* isolates in our study is rather low.

Resistance to tiamulin, a pleuromutilin, with a MIC value > 2 mg/L was observed in six *S. cohnii* and all *S. urealyticus* isolates. This antimicrobial is only licensed for use in pigs and poultry in Germany, but not in cattle. Pleuromutilin resistance may be mediated by the *cfr* or *vgaA/E* genes in staphylococci but these genes were not detected in the isolates of our study. Most likely, *S. cohnii* and *S*. *urealyticus* isolates harbor unidentified pleuromutilin resistance genes that are not covered by the NCBI amrfinder database, which is mostly based on sequence data from *S. aureus*. Accordingly, resistance to tiamulin could not be attributed to any predicted AMR gene in a study of NAS on dairy farms^38^. Moreover, rifampicin resistance according to EUCAST ECOFFs for coagulase negative staphylococci (MIC ≤ 0.064 mg/L) was detected in eight *S. cohnii* isolates and, with regard to the EUCAST ECOFFs for *S. aureus* (≤ 0.016 mg/L), in all *S*. *cohnii* and *S*. *urealyticus* isolates in our study. Rifampicin resistance may be mediated by point mutations in the *rpoB* gene^39^. Due to the limited data in public databases with respect to *rpoB* genes from *S. cohnii* or *S*. *urealyticus* as well as to antimicrobial resistant *S. cohnii* or *S*. *urealyticus* isolates in general, it is difficult to link mutations in the *rpoB* gene of the isolates from our study to the manifestation of rifampicin resistance.

In our study, resistance to trimethoprim was detected in *S*. *urealyticus* isolate SC4 with a very high MIC value (> 32 mg/L), whereas *S*. *cohnii* isolates SC3, SC8, SC10 and SC13 showed resistance with lower MIC values of 4–8 mg/L. Most likely, the high level trimethoprim resistance of *S*. *urealyticus* isolate SC4 was mediated by the *dfrK* gene. Since several strains harboring the *dfrC* gene did not show resistance to trimethoprim, this gene was apparently nonfunctional with respect to trimethoprim resistance in our isolates. The authors of one study described a physical linkage of the *dfrK* and *tetL* genes^40^, which might also be the case for *S*. *urealyticus* isolate SC4 in our study. However, *S*. *urealyticus* isolate SC11 harbored *tetL* without *dfrK*.

Only one *S*. *cohnii* isolate, SC9, might have been resistant to fosfomycin as predicted according to the presence of the *fosB6* gene. In clinical MRSA isolates from a Chinese hospital, the *fosB6* gene was located on a small plasmid and it was associated with a high level fosfomycin resistance (MIC > 256 mg/L)^41^.

Detection of *qacG/H* genes, which encode multidrug efflux pumps, was exclusively shown for the six *S*. *urealyticus* isolates. Qac efflux pumps may mediate resistance to disinfectant agents, including quaternary ammonium compounds, intercalating dyes and some antibiotics^42^. A widespread distribution of *qac* genes among staphylococci of bovine and caprine origin was shown in a study from Norway^43^. Moreover, another study reported *qacA/C* genes in NAS isolates from veal calves^34^. However, reports about *qacG/H* genes in NAS and in particular in *S. cohnii* or *S*. *urealyticus* isolates from livestock are rare and the first description of a *qacG* gene in livestock-associated MRSA sequence type 398 was not until 2015^44^.

The prediction of AMR genes and the phenotypic resistance to several beta-lactam antibiotics disagreed in 14/15 isolates. The acquisition of a SCC*mec* element appears to be rare in the analyzed *S. cohnii* isolates and was not observed in *S. urealyticus*. Only one *S*. *cohnii* isolate (SC7) harbored the *mecA* gene, located on a SCC*mec* type V. The SCC*mec* element type V was reported to be common in MRSA from dairy farms^26,33,37^. In our study, the *mecA* gene harboring *S. cohnii* isolate showed resistance to all tested beta-lactam antibiotics. Interestingly, all other isolates, in which *mecA* or *mecC* genes were missing, also exhibited a reduced susceptibility to cefotaxime (MIC 4–16 mg/L), ceftazidime (MIC 16–64 mg/L), cefoxitin (MIC 4–8 mg/L), temocillin (MIC > 128 mg/L) and penicillin (MIC 0.25–0.5 mg/L). In contrast, these isolates were susceptible to the beta-lactam antibiotics oxacillin, ampicillin, cefepime, meropenem, ertapenem and imipenem. The inconsistent resistance pattern indicates a different mechanism than the *mecA*/*mecC* gene transmitted mechanism, which results in resistance to virtually all beta-lactam antibiotics, as also determined for isolate SC7 in our study. The *mecA*/*mecC* gene mediated resistance is based on a modification of PBP2. Likewise, PBPs of the isolates in our study showed divergence from the known *S. aureus* PBPs. The amino acid sequence identities to the reference *S. aureus* PBP1, PBP2 and PBP3 were only 70–80%. It is possible that the differences in the amino acid sequences are associated with a structural difference in the protein, which may have lowered the binding affinity for some of the tested beta-lactam antibiotics. Since there is very little information on *S. cohnii* or *S*. *urealyticus* available in public databases and only sparse data regarding antimicrobial susceptibility of *S. cohnii* or *S*. *urealyticus* were published thus far, the possible function of the divergent PBPs in beta-lactam resistance is currently difficult to prove. The focus of our study and the respective methodological approach was the investigation of antimicrobial resistant *S. cohnii* and *S*. *urealyticus* isolates from dairy farms and thus no beta-lactam susceptible isolates were obtained for the necessary comparison of the AMR sequence data. However, one previous genomic study also conducted antimicrobial susceptibility testing with a *S*. *cohnii* strain^11^. This strain was susceptible to cefoxitin according to VITEK2 testing and also exhibited divergent PBPs, as did the isolates from our study (data not shown). Therefore, it is questionable whether the divergent PBPs of the *S*. *cohnii* isolates in our study triggered the partial beta-lactam antibiotic resistance. A borderline oxacillin-resistance in *S. aureus* isolates due to modified PBPs was previously suggested in a review about methicillin resistance in staphylococci^45^. Moreover, studies recently reported borderline cefoxitin- and oxacillin-resistant *S. aureus* isolates with substituted PBP amino acid sequences^23,46,47^. However, in both cases, the borderline resistance was associated with the hyperproduction of the BlaZ beta-lactamase. In our study, all *mecA*/*mecC* negative *S*. *cohnii* and *S. urealyticus* isolates were susceptible to oxacillin and the beta-lactamase encoding *blaZ* gene was not detected in any of these isolates. In addition, the susceptibility to ampicillin clearly indicates a lack of beta-lactamase expression, since this would have led to a reduced susceptibility to ampicillin. Moreover, the combination of cefotaxime or ceftazidime with clavulanic acid, a beta lactamase inhibitor, only marginally affected the susceptibility to the respective cephalosporins and did not lead to a susceptible wildtype phenotype. According to the NCBI Nucleotide database, *blaZ* genes may be present in *S*. *cohnii* isolates. However, further in depth analysis of *S*. *cohnii blaZ* genes using NCBI BLAST revealed that the sequences only show low identities (< 80%) to any other published staphylococcal *blaZ* gene. Along with the *mecA* and *mecC* gene, the *femA* and *gdpP* genes were associated with beta-lactam antibiotic resistance^23^ and both genes were detected in the isolates of our study. In general, however, data availability regarding AMR and its respective genes in *S*. *cohnii* and *S*. *urealyticus* is extremely limited and for this reason it is unclear how reliable the annotation and function of the so far published *S*. *cohnii blaZ* genes is. Due to the lack of comparable public data from susceptible and non-susceptible strains, the association of AMR with specific gene alterations currently cannot be proven.

Our study illustrates that the prediction of AMR phenotypes by genomic approaches in the case of rarely investigated staphylococcal species such as *S*. *cohnii* and *S*. *urealyticus* needs further research and improvement of established gene databases. Moreover, as already shown for other NAS species^20–22^, the specificity of cefoxitin testing for the detection of *mecA* or *mecC* genes and respective methicillin resistance in *S*. *cohnii* and *S*. *urealyticus* seems to be quite low. As reported for other *Staphylococcus* spp., oxacillin testing may be more reliable, but this needs to be confirmed on a larger strain collection.

In conclusion, our findings provide further impetus for the investigation of antimicrobial resistance in NAS, since these organisms have been thus far largely overlooked in this respect.

## Methods

### Ethical statement

Ethical review and approval was not required for the study because sampling of quarter milk samples was carried out in accordance with German legislation within the framework of diagnostic investigations on the dairy farms. No ethical approval from the Institutional Ethics Committee or the National Animal Experimentation Council was required. Samples were collected by a trained veterinarian with consent from the owners of the animals.

### Sample collection and whole-genome sequencing

For this study, 15 presumptive *S*. *cohnii* isolates, which were retrospectively identified as nine *S*. *cohnii* and six *S*. *urealyticus* isolates^5^, were selected for the analysis of antimicrobial resistance potential. The isolates were obtained from quarter milk samples of cows during a sampling campaign in 2018 and 2019 on twelve dairy farms from nine German federal states. Farms had been pre-selected based on a history of MRSA detection in the dairy cow herds^26^. The isolation procedure was based on a two-step selection with cefoxitin-containing media (3.5 and 4 mg/L). Along with MRSA, cefoxitin-resistant NAS were also detected by the isolation procedure and identified by MALDI-TOF analysis as described previously^26^. For evaluation of within farm genotypical differences, two independent *S. cohnii* isolates were analyzed from two farms. DNA from one 1 µl inoculation loop filled with colonies was extracted using the Qiagen DNeasy Blood and Tissue Kit (Qiagen, Germany) according to the manufacturer´s protocol modified by a 10 µl lysostaphin addition to the lysis buffer. The DNA library was prepared using an Illumina Nextera DNA Flex kit (Illumina Inc., USA) and the 150 bp paired-end sequencing run was performed on an Illumina NextSeq 500 instrument.

### Bioinformatic analysis

Raw Illumina reads were trimmed and de novo assembled with the in-house developed Aquamis pipeline (https://gitlab.com/bfr_bioinformatics/AQUAMIS/), which implements fastp^48^ for trimming and shovill (based on SPAdes) (https://github.com/tseemann/shovill) for assembly. Furthermore, it performs mash (v 2.1) for reference search^49^ as well as quast (v 5.0.2) for assembly quality control^50^. The assembled sequences were submitted to NCBI under the BioProject PRJNA641762. ANI search of NCBI revealed values of ≥ 96% for all isolates. Phylogenetic typing of isolates was performed using TYGS (https://tygs.dsmz.de/)^51^. Bacterial characterization was conducted with the in-house developed Bakcharak pipeline (https://gitlab.com/bfr_bioinformatics/bakcharak), which implements ABRicate (https://github.com/tseemann/abricate) for screening of antimicrobial resistance genes using the NCBI amrfinder database^52^. SCC*mec*-types were predicted using the software tool SCCmecFinder 1.2 from the Centre for Genomic Epidemiology (https://cge.cbs.dtu.dk/services/). Moreover, PBP1-3 genes of four annotated *S. cohnii* complete circular genomes (NCBI accession numbers UHDA01000001.1, CP027422.1, CP019597.1, CP033735.1), which were retrieved by NCBI Nucleotide search for “penicillin binding protein” and “*Staphylococcus cohnii*”, were extracted into an in-house database and *S. cohnii/S. urealyticus* assembled sequences from our study were searched by NCBI blastn against these known PBP genes. Translation of DNA to amino acid sequences and alignment to PBP1-3 of *S. aureus* extracted from NCBI as well as among the different PBPs of the detected *S. cohnii/S. urealyticus* isolates was conducted using MEGA X version 10.1.7 and ClustalW. A neighbor-joining phylogenetic tree was subsequently calculated comparing the PBP1-3 amino acid sequences from *S. aureus* and *S. cohnii/S. urealyticus* isolates. In addition, presence of the *mecB* (NG_047954.1), *mecC* (NG_047955.1), *mecD* (NG_054960.1), *blaZ* (KR270450.2), *femA* (AF145333.1), *femB* (GQ284649.1), *femC* (AP018376.1), *femD* (Y09570.1), *gdpP* (MF071106.1), *mgrA* (AP017922.1) and *mprF* (HM140977.1) genes was checked by individual NCBI BLAST against *S*. *aureus* or *Macrococcus caseolyticus* (*mecB* and *mecD*) reference genes.

### Antimicrobial susceptibility testing

Antimicrobial susceptibility testing was performed by broth microdilution according to the EUCAST and CLSI guidelines (ISO 20776-1:2006 or CLSI M31-A3). It was carried out using a standardized antibiotic panel (EUST scheme) that is recommended for use in all member states of the European Union for resistance monitoring on staphylococci from livestock and food^53^. Moreover, the standardized scheme was extended by a larger number of beta-lactam antibiotics as well as in combination with the beta-lactamase inhibitor clavulanic acid for analyzing resistance to beta-lactams in more detail. For interpretation of MIC of the individual isolates, the EUCAST ECOFFs for *S. aureus* and coagulase negative staphylococci were used, since specific MIC values for *S*. *cohnii* are not available (Tables 1, 2). For quality control of resistance testing, the *S. aureus* isolates ATCC 29213 and ATCC 25923 were used.

## Data Availability

The assembled sequences of all isolates in our study are deposited in NCBI under the BioProject PRJNA641762.
